# Personalized Lifestyle Intervention and Functional Evaluation Health Outcomes SurvEy: Presentation of the LIFEHOUSE Study Using N-of-One Tent–Umbrella–Bucket Design

**DOI:** 10.3390/jpm12010115

**Published:** 2022-01-15

**Authors:** Joseph J. Lamb, Michael Stone, Christopher R. D’Adamo, Andrey Volkov, Dina Metti, Lucia Aronica, Deanna Minich, Michelle Leary, Monique Class, Malisa Carullo, Jennifer J. Ryan, Ilona A. Larson, Erik Lundquist, Nikhat Contractor, Brent Eck, Jose M. Ordovas, Jeffrey S. Bland

**Affiliations:** 1 Personalized Lifestyle Medicine Center, Gig Harbor, WA 98332, USA; mstone@ashlandmd.com (M.S.); dinametti@plmc.com (D.M.); 2 Office of Personalized Health and Well-Being, Medical College of Georgia, AU/UGA Medical Partnership, Athens, GA 30606, USA; 3 Institute for Functional Medicine, Federal Way, WA 98003, USA; cdadamo@som.umaryland.edu (C.R.D.); deannaminich@hotmail.com (D.M.); monique@thecffm.com (M.C.); 4 Center for Integrative Medicine, University of Maryland, Baltimore, MD 21201, USA; 5 Bennet Data Sciences, San Diego, CA 92107, USA; andrey@bennettdatascience.com; 6 Metagenics, Inc., Aliso Viejo, CA 92656, USA; laronica@stanford.edu (L.A.); MalisaCarullo@metagenics.com (M.C.); ilonalarson@metagenics.com (I.A.L.); nikhat.contractor@amway.com (N.C.); BrentEck@metagenics.com (B.E.); 7 Department of Medicine, Stanford Prevention Research Center, Stanford University, Stanford, CA 94305, USA; 8 Human Nutrition and Functional Medicine, University of Western States, Portland, OR 97213, USA; 9 Vida Integrated Health, Seattle, WA 98112, USA; mleary@thinkvida.com; 10 The Center for Functional Medicine, Stamford, CT 06905, USA; 11 Helfgott Research Institute, National University of Natural Medicine, Portland, OR 97201, USA; jryan@nunm.edu; 12 Personalized Lifestyle Medicine Center, Aliso Viejo, CA 92656, USA; ErikLundquist@plmc.com; 13 Jean Meyer USDA Human Nutrition Center on Aging, Tufts University, Boston, MA 02111, USA; jose.ordovas@tufts.edu; 14 Personalized Lifestyle Medicine Institute, Bainbridge Island, WA 98110, USA; jeffbland@plminstitute.org

**Keywords:** health, quality of life, chronic disease, personalized, P4 medicine, precision medicine, functional medicine, personalized lifestyle medicine, functional assessment, N-of-one, methodology, single-subject analysis, diet, nutrition, nutrigenomics, nutritional supplementation, behavioral change, genomics

## Abstract

The working definition of health is often the simple absence of diagnosed disease. This common standard is limiting given that changes in functional health status represent early warning signs of impending health declines. Longitudinal assessment of functional health status may foster prevention of disease occurrence and modify disease progression. The LIFEHOUSE (Lifestyle Intervention and Functional Evaluation-Health Outcomes SurvEy) longitudinal research project explores the impact of personalized lifestyle medicine approaches on functional health determinants. Utilizing an adaptive tent–umbrella–bucket design, the LIFEHOUSE study follows the functional health outcomes of adult participants recruited from a self-insured employee population. Participants were each allocated to the tent of an all-inclusive N-of-one case series. After assessing medical history, nutritional physical exam, baseline functional status (utilizing validated tools to measure metabolic, physical, cognitive, emotional and behavioral functional capacity), serum biomarkers, and genomic and microbiome markers, participants were assigned to applicable umbrellas and buckets. Personalized health programs were developed and implemented using systems biology formalism and functional medicine clinical approaches. The comprehensive database (currently 369 analyzable participants) will yield novel interdisciplinary big-health data and facilitate topological analyses focusing on the interactome among each participant’s genomics, microbiome, diet, lifestyle and environment.

## 1. Introduction

Many chronic diseases are related to lifestyle and aging. In the United States, approximately 50 percent of the population suffers from a chronic disease. While contributing to 86 percent of healthcare costs [1], the human costs are immeasurable and require new approaches to address these potentially preventable lifestyle issues.

Advances in science and technology have led to a significant revision and modification of the medical models in practice today. Hood [2] has suggested that the best medicine should value four principles−medicine should be personalized, predictive, preventive and participatory. This “P4 Medicine” is personalized and patient-centered, focusing on the person and not the disease a person may have. It will be predictive in seeking to identify the preclinical trend/decline towards illness before the onset of symptoms that herald the loss of function and health. It will be preventive as the information gathered offers opportunities to modify these trajectories towards illness. Finally, it will be participatory as individuals will be intimately involved in gathering data to identify trends and apply lifestyle measures to improve their lives.

There is a significant unmet need to identify the value of personalized lifestyle interventions for improving functional health outcomes. Whereas disease is well understood from traditional pathology-based indices, health is less easily defined. Historically, healthy has been the default term applied to an individual who is not recognized as having a disease. This definition of health as the absence of disease has resulted in the delivery of health and wellness as often lying outside the purview of medicine.

An evolving definition of health recognizes that health is directly related to the functional capacity of the individual. Changes in functional health status over time can represent early warning signs of later disease. Functional capacity may well be categorized in four assessment areas: metabolic, physical, cognitive and emotional Assessment of these four areas reflect the organ reserve sustaining the physiologies reflected in the Functional Medicine Matrix, specifically assimilation, defense and repair, energy, structural integrity, biotransformation and elimination, communication and transport. Our capacities to work, think, communicate and to feel are quantifiable by both qualitative and quantitative metrics exploring the four areas [3,4].

**Figure 1:**
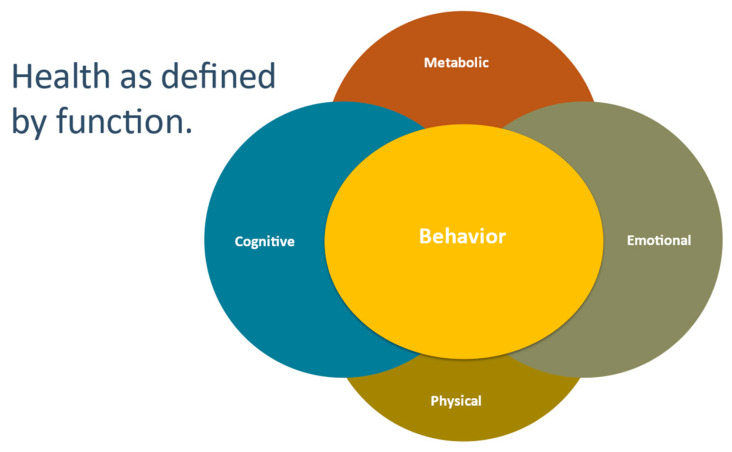
Five-functions behavior represents the outward expression of improvements recognized in metabolic, physical, cognitive and emotional functional capacities.

Achievement of health through a P4 model [5] assumes the ongoing adoption of therapeutic behavioral changes by the participant. It is acknowledged that failure to achieve meaningful and lasting change is an obstacle to health promotion. Recognizing this, we realized that there is a crucially important fifth function, behavior, as it represents the outward expression of improvements recognized in metabolic, physical, cognitive and emotional functional capacities (Figure 1). We believe that evaluating functional metrics may be the most direct and efficacious means by which to quantify health and create programs for lasting behavioral change.

Creating health through evaluation of function and an understanding of disordered pathophysiology requires practitioners to identify core variables while utilizing a diagnostic approach different than the standard development of a differential diagnosis list or even a more targeted split into most important diagnoses and most likely diagnoses. Indeed, evaluating a large dataset, inclusive of genomic and microbiome interactions beyond the typical biomarkers, presents challenges even in generating these standard approaches. In LIFEHOUSE, we employ the model of P4 medicine, personalized lifestyle medicine and functional medicine to address this need. In a 2013 article, Hood and colleagues wrote the following:

“Ten years ago, the proposition that healthcare is evolving from reactive disease care to P4 was regarded as highly speculative. Today, the core elements of this vision are widely accepted and have been articulated in a series of recent reports by the US Institute of Medicine…. It will provide the basis for concrete action by consumers to improve their health as they observe the impact of their lifestyle decisions [6]”.

In another article published that same year, Hood describes P4 medicine in terms of individual engagement, behavioral changes to improve health outcomes and personalization of diet and lifestyle [7]. As first described by Bland and Minich in 2013, personalized lifestyle medicine is a concept and clinical approach that utilizes patient-centered information [8]. Whereas population-based studies deliver conclusions based on statistics describing from group norms, personalized lifestyle medicine is focused on gathering and interpreting patient-specific information to understand function in an N-of-one manner. The concept of personalized lifestyle medicine emerged, in part, because an approach to disease-risk reduction based on population data alone lacks efficacy in modifying health behaviors or optimizing health and wellness. For more than 20 years, a field of medicine that is uniquely dedicated to studying function has been growing and gaining prominence among professional communities. functional medicine was first developed as a clinical approach in 1991 [9]. After two decades of evolution and refinement due to active application in clinical practices worldwide, functional medicine has demonstrated itself to be an actionable and effective approach to delivering personalized lifestyle medicine to patients. The foundational core tenets of functional medicine include being patient-centered and systems biology-focused; recognizing the importance of gene–lifestyle–environment interaction; promotion of organ reserve; and respect for the philosophy that health is a positive vitality and not simply the absence of disease [10]. functional medicine practitioners recognize that health results from the relationship between form and function in each unique individual [11]. In the clinical setting, practitioners often focus on recognizing antecedents, triggers and mediators that can result in functional changes affecting health status.

New and emergent tools and technologies can be utilized to measure and monitor functional health status in an ongoing manner, leading to opportunities for early intervention and improved patient outcomes prior to the onset of disease. The LIFEHOUSE (Lifestyle Intervention and Functional Evaluation−Health Outcomes SurvEy) longitudinal research project is designed to evaluate the impact of a personalized lifestyle intervention program on functional health determinants. Utilizing an adaptive tent–umbrella–bucket (TUB) design, the LIFEHOUSE study follows the functional health outcomes of a group of adult volunteers.

## 2. Materials and Methods

LIFEHOUSE is a series of ongoing clinical surveys; and as such, the tent and the individual umbrellas with their respective buckets have been reviewed and approved by the Aspire Independent Review Board (IRB) (Santee, CA, USA), now a member of the Western-Copernicus Group IRB. Our survey has been registered with ClinicalTrials.gov as NCT04005456.

### 2.1. Research Goals Addressed by the LIFEHOUSE Survey Design

Within this structure, we have developed a comprehensive multi-component data set of information from all participants in a relational structure that allows integration of genomic, microbiome, biometric, wearable device, phenomic, lifestyle, environmental (exposome), psychosocial and nutritional information. Our data set provides the opportunity to assess the effectiveness of current and new analytic techniques that enable practitioners to manage and evaluate this flow of information so it can be utilized to facilitate creation of health and maintenance of lifestyle change by the patient.

We desire to identify the distinction between health as the simple absence of disease and as the creation of a journey to wellness by developing tools to evaluate an individual’s functional capacities. To enable fruitful interventions and behavioral change, practitioners need to suggest effective personalized lifestyle medicine interventions. Effective measures should be those that both increase functional capacity and are meaningful to the patient. Measurements (such as grip strength, evaluations of balance and cognition) and utilization of a broad range of questionnaires facilitate a functional baseline assessment, a metric by which therapeutic interventions can be judged for their efficacy. Additionally, we will explore the data set to develop an understanding of determinants of functional fitness and resiliency characteristics and their relationship to composite polygenic risk scores.

The annual physical examination is a core component of the medical model. Information obtained during this once-yearly interaction between clinician and patient ideally would provide insight into an individual’s health status. Mehrotra and Prochazka address the failure of the routine health physical in terms of its ability to reduce both illness and premature mortality from non-communicable chronic diseases (NCD) [12]. Krogsbill et al. stated the following: “Health checks were not associated with lower rates of all-cause mortality, mortality from either cardiovascular disease or cancer. Health checks may be associated with more diagnoses and more drug treatment [13].” Performing a detailed physical examination is a challenge during a 15-min primary or specialty care visit, particularly in an era when confirmation by laboratory biomarkers and readily accessible imaging techniques (including magnetic resonance imaging, computed tomography and ultrasound) is readily available. However, what is nonstandard and not easily accessible to the practicing practitioner is the ability to order a detailed appraisal of the nutritional status and the functional capacity of the patient. Hence, identifying features of the exam that suggest the necessity for further diagnostic testing or indeed are indications for dietary modification or nutritional supplementation would be valuable. The components of the physical exam were selected due to their ability to reflect disorder and imbalances in the basic components of the functional medicine matrix and to indicate potential nutritional deficiencies. Certain components, including oral and nasal mucosae, and skin are particularly relevant given their ability to reflect recent changes. While certain relationships (such as that between essential fatty acid deficiency and hyperkeratosis pilaris) are already established, confirmation in our data set will be sought. New associations, between exam findings and the broad range of information in our data set, will be explored by utilizing principal component analysis.

Our clinicians faced the challenge of dealing with large data sets during their sequential 30–45 min visits; initially focusing on obtaining a detailed 45-component nutritional exam (Table 1) and progressing to a review of history coupled with tent–umbrella–bucket assignments and creation of personalized lifestyle medicine intervention programs. These programs were protocol driven (quite prescriptive at the bucket level and more permissive at the tent level, utilizing biomarker-driven adaptive rules for basic nutritional supplementation) and included dietary, exercise, cognitive behavioral, lifestyle and nutritional supplement recommendations. Bringing organization to the current pattern-recognition filters by identification of key components (confirmation of known associations) and identification of new relationships will facilitate assignments in the next steps of our work and in assisting practitioners in identifying core driver variables that result in actionable interventions that are associated with potentially durable improvement in functional health outcomes. Development of our unique data set offers an opportunity to apply principal component analysis and Bayesian techniques to create tools that standardize these filters. As it becomes understood that a population-based approach to assessing NCD-related risk has had limited success in reducing the global burden of chronic disease [14], healthcare systems worldwide are now faced with a new challenge: to innovate approaches that will measurably deliver improved health outcomes to the individual. With primary care guidelines also in a state of flux, this new system must be created with several imperatives in mind, including ways to engage the individual in health objectives using a different context from the traditional disease risk-reduction model. Indeed, questions and concerns have even been raised about the future of primary care as a specialty, and some researchers have suggested this discipline cannot survive in its present form given the increased demands associated with managing patients with chronic diseases and the constraints of the present structure as primary care providers struggle to provide effective care [15]. New methods, including group medical visits, supportive educational materials, counselling by lifestyle educators and effective use of time during clinical visits to engage participants, have been explored during LIFEHOUSE. The utility of these methods has been evaluated by assessing compliance, attendance and participant feedback.

A primary interest of ours was to demonstrate the efficacy of personalized lifestyle medicine. LIFEHOUSE as an adaptive tent–umbrella–bucket N-of-one design is uniquely suited to explore this important clinical question, by having longitudinal measurements of participants receiving intervention, with the potential to comparing to their baseline, as a control. In particular, our goal was to determine the impact of a personalized lifestyle medicine intervention program on wellness by exploring the five categories of function: metabolic, physical, cognitive, emotional and behavioral.

### 2.2. Participants

The target population was generally healthy adults. Approximately 400 participants, male and female, ages 18–80, from a single company’s insured employee population were recruited in the N-of-one IRB-approved study; this population represents the tent. Exclusion criteria were serious unstable illnesses inclusive of cardiac, hepatic, renal, gastrointestinal, respiratory, endocrinologic, neurologic, immunologic/rheumatological, infectious, hematologic and psychiatric diseases, such as unstable angina, recent myocardial infarction, viral hepatitis, cirrhosis, end stage renal disease, gastro--intestinal bleeding advanced chronic obstructive pulmonary disease, tuberculosis, thyrotoxicosis, Parkinson’s disease, acquired immunodeficiency syndrome, major depression, schizophrenia and ongoing addiction to controlled substances. At regular intervals, participants were reassessed using validated functional assessment tools and a variety of biometric and laboratory evaluations.

Our preliminary data consists of 369 participants who had met the minimal requirements for data analysis which was deemed to consist of initial clinical visit and baseline anthropometrics and serum biomarkers.

### 2.3. Tent–Umbrella–Bucket Assignment

**Figure 2:**
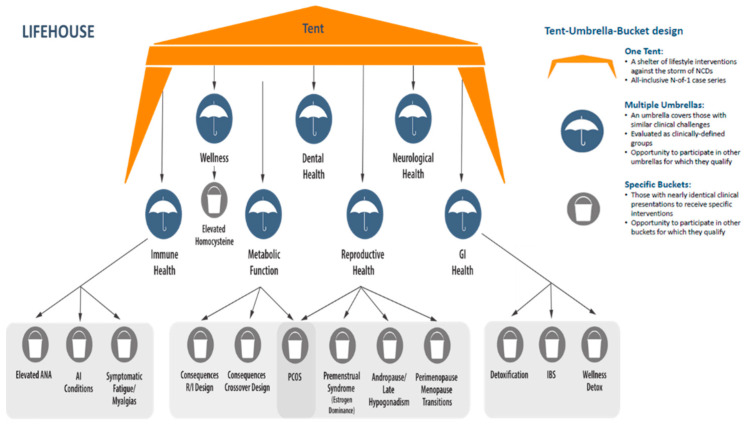
The unique tent, umbrella and bucket designations used in LIFEHOUSE allowed participants to be assigned to specific interventions under the umbrella and buckets, while still included in the overall N-of-one tent during periods of observation and inactivity. Abbreviations: gastrointestinal (GI) health, antinuclear antibody (ANA), autoimmune (AI), randomization/inclusion (R/I), polycystic ovary syndrome (PCOS, irritable bowel syndrome (IBS).

Many of the participants participated in a previous Aspire IRB approved protocol (“A Study to Examine Correlations Between Lifestyle Factors, Genomic Data, Physical Exam Findings and Biomarkers”). Upon completion of this 6-month observational period, participants were offered the opportunity to participate in LIFEHOUSE. At their first LIFEHOUSE visit, participants were enrolled in the overall N-of-one tent and were assigned to an umbrella and/or bucket if requirements were met. Assignment was determined by the clinician during visits. The structure of the TUB design is illustrated in Figure 2.

Participants only received one specific umbrella/bucket assignment at a time, and once completed, they re-entered the observation phase. If participants qualified for more than one umbrella/bucket assignment, they were later offered an opportunity to enter additional umbrella/bucket assignments. Inclusion criteria for umbrellas/buckets are described below.

1. The wellness umbrella includes participants with minimal physical complaints and broadly normal, yet perhaps not optimal biomarkers. Within this umbrella is the elevated homocysteine bucket, for individuals with elevated homocysteine levels (≥10.4 µmol/L). A level of ≥10.4 µmol/L was selected given our reference laboratory’s Cleveland Heart Lab (Cleveland, OH, USA) identification of this level as the upper limit of normal for women. It has been proposed and accepted in integrative medicine circles that a significantly lower level may be representative of optimal function. Serial evaluation of healthy individuals may clarify whether homocysteine is best thought of as a biomarker or a target for intervention in the creation of wellness [16].
2. The dental health umbrella includes participants with established dental disease, such as gingivitis, periodontitis, caries, painful teeth or fractured teeth, requiring active care.
3. The neurological health umbrella includes participants experiencing a broad range of neurological degenerative diseases, neurocognitive issues, and psychological/emotional complaints.
4. The metabolic health umbrella included individuals classified as desirable weight with markers of dysglycemia/dyslipidemia and classified as overweight/obese with and without markers of dysglycemia/dyslipidemia. The consequences of metabolic function/dysfunction bucket−crossover design compared three dietary and nutritional supplement programs (a ketogenic program, a high protein/high phytonutrient program and a Mediterranean style low glycemic load program. In the consequences of metabolic function/dysfunction bucket−randomization/inclusion design participants were randomized to one of three dietary and nutritional supplement programs (a ketogenic program, or a high protein/high phytonutrient program or a Mediterranean style low glycemic load program). Final assignments into applicable umbrellas or tubs reflected a joint decision between participant and clinician.
5. The gastrointestinal health umbrella was designed for individuals with conditions associated with issues of gastrointestinal health and of environmental toxicity or dysfunction involving metabolic transformation. Participants with irritable bowel syndrome (IBS) were enrolled in the IBS bucket. Generally healthy participants were eligible to enroll in the wellness detoxification bucket. Participants demonstrating significant health challenges were eligible to enroll in the detoxification bucket.
6. The immune health umbrella was designed for individuals with autoimmune/inflammatory conditions (excluding metabolic disorders/atherosclerosis). Assignment to the elevated anti-nuclear antibody bucket required elevated antinuclear antibodies (ANA) levels and preclinical symptomatology only. The autoimmune conditions bucket included participants with established autoimmune conditions (systemic lupus erythematosus, rheumatoid arthritis, inflammatory bowel disease and Hashimoto’s thyroiditis). The symptomatic fatigue and myalgias bucket enrolled participants with symptoms of persistent fatigue and myalgias.
7. The reproductive health umbrella was designed for men and women with conditions associated with reproductive and hormonal health (including fibrocystic breasts and/or increased breast cancer risk, endometriosis, testosterone deficiency and andropause/late onset hypogonadism, and prostate health). The perimenopausal and menopausal transitions bucket enrolled for women with symptomatic perimenopausal and menopausal transitions. The premenstrual syndrome bucket was created for women with premenstrual syndrome and alterations in estrogen/progesterone balance. The PCOS bucket is a unique bucket with enrollment criteria capturing the mixed metabolic dysfunction and reproductive hormonal disturbances typical of women diagnosed with PCOS and is thus shared between the metabolic health umbrella and the reproductive health umbrella.

### 2.4. Data Collection

#### 2.4.1. Baseline Data Collection

**Figure 3:**
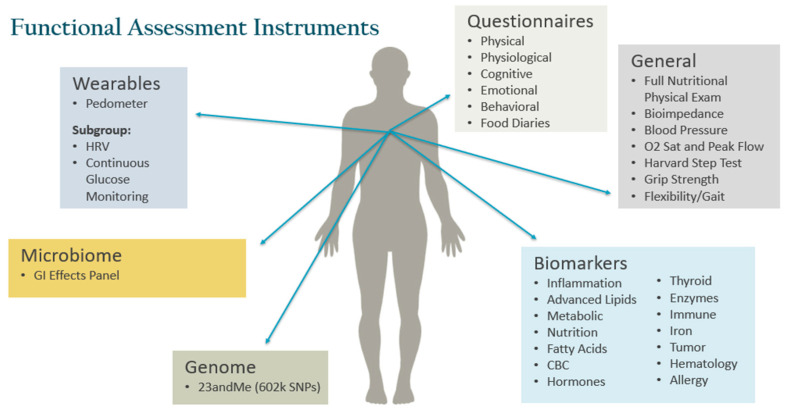
Functional Assessment Instruments for Metabolic, Physical, Cognitive, Emotional and Behavioral Function. Abbreviations: heart rate variability (HRV), gastrointestinal (GI), saturation (sat), complete blood count (CBC).

Baseline data collection was extensive and included detailed medical history, questionnaires, anthropometrics, nutrition physical exam, functional evaluations, microbiome analysis and single-nucleotide polymorphism assessments (Figure 3). Specialized testing was ordered in specific buckets and physical locations as per protocol. All participants signed an informed consent forms prior to data collection.

An 18-page history form was completed by all participants. Additional questionnaires included:

- Rand MOS SF-36 [17]
- Health Symptom Questionnaire
- PROMIS-43 Questionnaire [18]
- Depression, Anxiety and Stress Scale [19]
- Beck Depression Inventory [20]
- Beck Anxiety Inventory [21]
- PROMIS Anxiety SF [22]
- PROMIS Depression SF [23]
- PROMIS Sleep Disturbance SF [24]
- PROMIS Cognitive SF [24]
- VIA Strength Finder [25]
- University of Rhode Island Change Assessment (URICA) [26]

Assessment conducted during clinical visits included the following measures: anthropometrics, vitals, body composition and nutrition physical exam.

Phlebotomy was performed for both plasma and serum to measure:

- comprehensive metabolic panel
- chemistries, including gamma-glutamyltransferase (GGT), uric acid and osteocalcin
- markers of dysglycemia including insulin and HbA1c
- complete blood count
- nutritional markers, including 25-OH vitamin D3, homocysteine, ferritin, folate, magnesium, omega-3/omega-6 fatty acid profile
- advanced lipid panel, myeloperoxidase (MPO), oxidized low-density lipoprotein (Ox-LDL) and lipoprotein(a) (Lp(a))
- inflammatory markers, including high-sensitivity C-reactive protein (hs-CRP), antinuclear antibodies (ANA), anti-cyclic citrullinated peptide (Anti-CCP) and rheumatoid factor (RF)
- endocrine markers, including cortisol and comprehensive thyroid panel
- male and female sex hormones including pregnenolone, progesterone (women), dehydroepiandrosterone sulfate (DHEA-S), testosterone, dihydrotestosterone (DHT) (men), estradiol, follicle-stimulating hormone (FSH) (women), luteinizing hormone (LH) and sex hormone binding globulin (SHBG)
- immunoglobulin G (IgG) food allergy testing
- prostate-specific antigen (PSA) (men over 50 years old)

The biomarkers were routinely measured by Cleveland Heart Lab and Quest Diagnostics.

For microbiome assessment, stool was collected and analysis conducted by Genova Diagnostics, Asheville, NC. Saliva was collected for the assessment of genomic data by 23&Me, Sunnyvale, CA [27]. Selected participants had plasma banked for possible proprietary lipid mediator profiling analysis.

Functional status of all participants was tested at baseline and included (beyond the questionnaires mentioned above) forced expiratory volume [28], grip strength [29] and measures of flexibility/balance and observation of gait [30]. Some participants completed the Harvard Step Test to measure exercise capacity.

Specialized testing could be offered to participants based upon protocol rules and geographic location. This testing could include urinary toxic elements (Doctor’s Data, Inc., St. Charles, IL, USA), peripheral artery tonometry using the Endo-PAT device (Itamar, Inc., Atlanta, GA, USA), adrenal stress profile with awakening cortisol response (Genova Diagnostics, Asheville, NC, USA). Participants demonstrating compliance with protocol requirements received an Apple Watch, series 3 (Apple, Inc., Cupertino, CA, USA) for biomonitoring. Selected participants were asked to do continuous glucose monitoring and heart rate variability testing.

#### 2.4.2. Follow-Up Data Collection

Participants were seen by a study clinician at baseline and every six months during periods of active participation. Additional in-person visits were dictated by applicable tent, umbrella and bucket requirements. Virtual visits were conducted at 3-month intervals between the 6-month in-person visits. Additional virtual visits were scheduled to assess progress and acute adverse events. The principal investigator had latitude to order off-protocol laboratory testing to initiate evaluation of recognized laboratory and physical exam findings to facilitate further medical evaluation. Per protocol, history and questionnaires, anthropometrics, vitals, body composition, nutrition physical exam, laboratory biomarkers, microbiome analysis and specialized testing were repeated at regular intervals of 6 and 12 months.

### 2.5. Interventions

**Figure 4:**
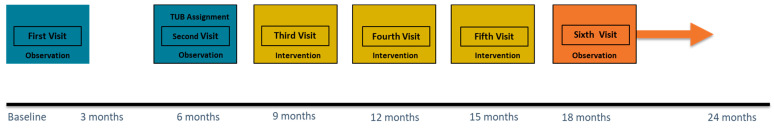
Timeline of study interventions and observation phase.

One hundred eighty-four participants were invited, after TUB assignment, to begin an active treatment phase. The majority of these participants were in the metabolic health umbrella (*n* = 83), the immune health umbrella (*n* = 72) or detoxification buckets (*n* = 18). Intervention programs were personalized within the scope of adaptively designed TUB design. These participants were followed for an average of 9 months before moving once again into an inactive observation phase (Figure 4). All participants are currently in an inactive observation phase, where participants entered a period of free-range behavior in which they are neither being monitored nor observed.

### 2.6. Data Management

Study data were either collected on case report forms (CRFs) designed for the study, directly entered in Microsoft (Redmond, WA, USA) Excel 2007 databases or received electronically as de-identified data directly or using secure file transfer protocol sites. Source documents included all recordings of observations or notations of clinical activities and all reports and records necessary for the evaluation and reconstruction of the clinical survey including use of an electronic medical record (EMR) (NextGen, Irvine, CA, USA). Data for CRFs was collected during participant visits, phone calls between participants and health care providers and from participant submissions (questionnaires and data logs).

### 2.7. Statistical Analysis

Methods for the analysis of N-of-one case series have been evolving quickly. A detailed discussion of these methods is available in a monograph on the conduct of N-of-one case series by the Agency for Health Quality and Research [31]. Senn is quoted in Offord [32] stating that it is important to use Bayesian techniques to distinguish between various sources of within- and between-patient heterogeneity. Dynamic expression modeling addresses the challenges of the role of time and the influence of the past on the future [33].

In LIFEHOUSE, the analyses are being performed on per-protocol data sets, including an intent-to-treat data set and a data set consisting of all participants who finish the survey. Individual analyses will be similarly conducted for each umbrella and bucket. Missing data will not be imputed. Subgroup analysis based upon gender, age, body mass index (BMI), presence/absence of defined biomarkers at baseline, readiness to change (based upon URICA questionnaire score), manufacturing versus corporate work settings, and compliance are being conducted.

As applicable, non-parametric tests, including the sign test and Wilcoxon signed-rank test, will be conducted. As our survey design features possible consecutive and sequential participation in the TUB and a rolling enrollment, applicable techniques to account for continuous outcomes, effects dependent on time, autocorrelation of measurements (both serial correlation and dynamic models) and carryover effects will be conducted. Standard estimation techniques, both paired and unpaired t-tests, were conducted as appropriate. Finally, principal component analyses were conducted. Primary data analysis was conducted by Bennett Data Sciences, San Diego, CA, USA.

## 3. Results

Table 2 presents the baseline characteristics of our 369 participants (those currently enrolled and with sufficient data for analysis). The participants had a mean age of 42 at study enrollment. The BMI of all participants averaged 27.4 ± 6.0 kg/m^2^. The corporate/sales group has a slightly lower BMI of 26.5 ± 5.1 kg/m^2^, compared with the manufacturing group, which had a BMI of 30.5 ± 7.4 kg/m^2^ (*p* < 0.001). BMI ranges in kg/m^2^ are 18.5–24.9 desirable; 25–29.9 overweight; ≥30.0 obese [34] Almost 66% of participants are female, which remained true for the corporate/sales group and dropped to 60% for the manufacturing group. Participants were primarily White 65%, while approximately 12% were Hispanic, 11% were Asian, 6% were African American, and 4% were Native American.

## 4. Discussion

To address a broad series of current therapeutic challenges, LIFEHOUSE is an adaptive, function-focused survey incorporating features of randomized, placebo-controlled trials to answer targeted questions while maintaining an N-of-one approach to focus on our broad themes of defining health by function, exploring efficacious models of behavioral change and support for a personalized lifestyle medicine approach in addressing the chronic diseases of lifestyle and aging.

An adaptive clinical trial is an innovative trial design that conserves both resources and time and allows for multiple interventions to be tested simultaneously with the goal of identifying “best practices” for individuals residing within specific clinical definitions or subgroups [35]. Adaptive clinical trials offer flexibility within the design of the study to personalize care for participants (e.g., nutritional supplementation dependent upon measured sufficiency or genomic uniqueness) and to provide participants with optimal care (adaptive arm allocations).

As illustrated by Kravitz et al. [31], advances have been made in trial design to facilitate the collection of subject-oriented outcomes. Advanced statistical techniques allow for aggregation of large N-of-one case series and collection of data in ways that are novel. N-of-one trial protocols offer an objective, efficient and cost-effective method of conducting such trials. Basket-and-umbrella trial [36,37] designs have been recognized as an effective design to approach these large N-of-one case series.

Although the use of the basket-and-umbrella trial design has been limited primarily to the evaluation of drugs used in oncology, the concept has tremendous potential for the study of personalized lifestyle medicine interventions. A variation of this type of study design has already been used successfully by researchers investigating the impact of lifestyle and behavior on frailty. The Doetinchem Cohort Study evaluated physical, cognitive, and emotional function in adults aged 40–81 years who were living with different degrees of frailty [38]. To understand the etiology of frailty, these study investigators concluded that multiple functional domains need to be evaluated and that lifestyle interventions should be personalized to meet individual functional needs.

When applied to a personalized lifestyle intervention trial, the N-of-one design would allow for the creation of a program tailored to each participant based on their individual metabolic, physical, cognitive, emotional and behavioral needs and determined through genomic analysis, medical history and data sets of metabolic, microbiomic, physical, cognitive, emotional, lifestyle and behavioral status. The aggregation and segmentation of data sets across the participant population can identify groups with similar clinical presentations yet segmented by genomic, microbiomic and functional uniqueness. N-of-one designs allow for this uniqueness to address, with personalized lifestyle medicine recommendations and clusters, these individuals according to shared similarities into several different subgroups (umbrellas, buckets) of the group at large (tent) to be analyzed. The design described here encourages the development of clinical guidelines based on these individual patient experiences analyzed through this lens of a TUB stratification.

Additionally, this approach also allows for the development of a nested family of case reports, which could potentially identify areas of health care in which personalization may positively impact clinical outcomes [39].

In 2004, the American College of Lifestyle Medicine (ACLM) was founded with the following stated purpose: “To educate, equip, and empower individuals to provide the information and resources that a patient needs to protect their health and fight chronic disease [40].” Widespread introduction of this concept at the clinical level has proven challenging. It is now well recognized that genetic, biological and behavioral aspects of health have a high degree of variability among humans [41]. From their experience and scholarship has emerged the perspective that successful management of chronic disease is a balance of art and science that comes together using a lifestyle-focused approach to primary care that can reverse global chronic disease epidemics [42,43].

Disease-risk reduction is closely linked to public health initiatives related to prevention. However, though based on inherent good intent, such efforts have limited success in terms of long-term behavior change. In addition, prevention is a metric that is difficult to measure at the individual level. How does an individual know, without ambiguity, that actions they took resulted in the prevention of a disease? While prevention cannot be easily quantified, improvement in function can be assessed, documented and tracked. For this reason, health status is likely better defined by function than by disease-risk reduction and prevention. Function results from the ongoing interaction of key personal (and highly unique) variables, including the following: genetics, age, gender, ethnicity, health history (both medical and personal), lifestyle, diet, stress patterns, activity, sleep, medication and dietary supplement use and environmental exposures. These variables represent opportunities to apply a systems biology approach to personalized lifestyle medicine.

In 2017, Bland, Minich and Eck co-authored an article describing an approach to improving individual health and wellness using systems biology and unique functional assessments to improve the effectiveness of lifestyle medicine [44]. Technology now provides the tools to collect data in ways not previously possible. Individuals collecting information on their genome (including their genetic predisposition to tolerate medications and respond to healthy lifestyle programs) may modify their lifestyle and therapeutic choices to optimize their wellness.

As noted above, function in an individual can be segmented into five subgroups for assessment: metabolic, physical, cognitive, emotional and behavioral function. Validated tests and/or questionnaires are available for all these subgroups. Tests to measure functional reserves may differ from those used to determine the presence of disease, but this does not diminish their value to both clinicians and patients. An example of highly useful functional assessment tools is continuous blood glucose monitoring, which permits the evaluation of lifestyle’s impact on glucose metabolism [45]. Similarly, cognitive function can be measured using tests available from the National Institutes of Health Toolbox [46], while physical function can be measured using a number of common resources designed to assess strength, endurance, flexibility, and balance [47,48,49,50]. Lastly, a comprehensive portfolio of validated questionnaires is readily available to measure behavioral/emotional function [51,52,53,54]. Additionally, data on lifestyle, including food consumption (at the caloric, macronutrient and even micronutrient level can be collected for each meal), exercise and sleep quality can be gathered electronically. 

As has been noted by Price et al. [55], a significant challenge to the effective use of these complex sets of individual patient data is how to define the boundaries between disease, average health and optimal wellbeing. To meet this challenge, compiling and analyzing collections of de-identified, detailed patient histories, questionnaires regarding symptoms and general condition and associated objective findings (genomic data, vital signs, and physical exam and laboratory biomarkers) will theoretically identify these boundaries and will facilitate the deliverance of functional medicine-congruent lifestyle interventions. Comprehensive data collections on each subject evaluated in aggregate provide a diversity of unique markers that can be statistically probed to identify patterns that predict wellbeing and, perhaps, an individual response to lifestyle interventions.

Aggregation of such functional assessments has several advantages over a traditional disease-risk evaluation. By mapping the functional capacity of an individual, the patient gains access to quantitative tracking tools to evaluate how lifestyle changes influence personal function. At the same time, clinicians now have the robust information needed to personalize and manage therapeutic recommendations. One goal of the approach described here would be the high level of patient engagement that may come with the ability to self-track intensely personal metrics. The definition of health is very personal, and this approach allows the individual to determine which functional indicators they are both most interested in and willing to modify. Overall, this concept moves lifestyle medicine intervention from a population-based, risk-factor-reduction model to one that is focused on individual function and performance.

In a clinical observational research study using an N-of-one tent–umbrella–bucket design, it is logical to use P4 medicine, personalized lifestyle medicine and functional medicine methodology as core clinical tools for intervention. As functional medicine has been successfully utilized in the implementation of personalized lifestyle medicine programs [4,56], it is uniquely suited to the study of quantifying health and wellness through the use of the five functional categories described above.

Thus far, we have described the many strengths of the LIFEHOUSE design. However, our initial work within the scope of the survey has also demonstrated some of the weaknesses. Our study clinicians and study staff have faced several challenges. The primary challenge was the struggle to manage the wealth of data collected. Due, early on, to the lack of integrated data management systems, data were not always immediately accessible to the clinicians as they made care decisions within the bounds of the protocol rules. Additionally, study clinicians struggled with the tension between collecting data and using the data to assist participants in making personalized lifestyle medicine therapeutic choices in a meaningful way. We additionally have found that our participants and study clinicians/staff in many cases have not shared similar goals; presented with complex interventions detailed by study protocols, participants not infrequently have struggled with maintaining compliance, if not interest. The wealth of choices within the latitude of the tent and the individual umbrellas also created challenges for the staff to coordinate the delivery of care.

In looking at our next steps and in recognizing our design’s strengths and limitations, we have been engaged actively in process improvements in our data collection tools and creating new tools to foster behavioral change which will be introduced in future intervention phases. We are currently writing the protocol for LIFEHOUSE 2.0, a data registry survey evaluating the personalized lifestyle medicine experience in the two Personalized Lifestyle Medicine Centers.

LIFEHOUSE is a living, evolving study that will continue to produce a wealth of exploratory opportunities related to our key research goals. This work currently has resulted in several planned papers and will be continuing. New opportunities will be created as we modify the survey design and increase both the diversity and amount of data available for analysis. We welcome discussion with interested potential research partners.

## 5. Conclusions

Every individual has unique and ever-changing patterns in five functional capacities: metabolic, physical, cognitive, emotional and behavioral. New personalized and adaptive research approaches are needed for investigators to understand the impact of gene-environment interactions on health as measured by function and to demonstrate the value of interventions suggested by these interactions.

LIFEHOUSE represents an innovative approach to studying functional health outcomes in a personalized lifestyle medicine program using a tent–umbrella–bucket protocol. New data management and analysis methods provide tools to determine areas of similarity among different individuals that will allow for valid conclusions to be drawn from the data. It is important to note that LIFEHOUSE addresses multiple important clinical questions, including “What is Health?” and “How can we promote Health effectively and equitably?” Our design aims to provide data demonstrating the effectiveness of a P4 medicine model (personalized, predictive, preventive and participatory). Combining a functional medicine intervention model in the P4 medicine context represents a unique approach to examining the impact of a systems biology formalism and the delivery of personalized lifestyle medicine on health outcomes. LIFEHOUSE, as a thriving, broad series of research initiatives, is expected to reveal much about factors that may be key drivers of effective behavioral change and positive functional health outcomes, about the clinical application of personalized lifestyle medicine and about the successful execution of a large-scale adaptive N-of-one protocol.

## Figures and Tables

**Table 1:** Components assessed for the Nutrition Physical Exam (NPE).

Nutritional Assessment	Variable
body composition findings	normal
	abnormal body mass index
	elevated waist circumference
	elevated waist-to-hip ratio
	abnormal body fat
body type	normal
	cachectic
	underweight
	skinny fat
	gynoid
	android
blood pressure	normal
	hypotensive
	hypertensive
	symptomatic
hair distribution	normal
	alopecia
	androgenic alopecia (female)
tongue	normal
	size
	shape
	color
	coating
	taste bud distribution and prominence
	fissuring
	ankyloglossia
	lesions
	tongue varicosities
	Wharton duct blocked
	saliva ph (<6.8/>6.8)
gums	normal
	lesions
	gum line color
	bruising
	lesions
	macules
	tenderness
	gum line darkening
	gingivitis
	periodontal disease
	hyperplasia
teeth	healthy—no restorations
	missing teeth
	tooth attrition or abrasions
	silver/mercury filling
	silver next to gold
	bridges/dentures
	periodontal ligament pain
	enamel marks (dysplasia)
	discoloration (fluorosis)
	plaque (tartar)
jaw movement	symmetric
	asymmetric
	auscultated crepitus or click
	pain
	mouth opening
lips	normal
	dry, cracking
	angular cracks or sores
	ulcerations
	fissures
	perioral rash
	loss of lip borders
	lesions
	edema, angioedema
	piercing
soft palate, hard palate, tonsils, pillars	normal
	cleft or oropharyngeal defects
	boney lesions
	soft palate
	lesions
	tonsils
	breath
buccal mucosa	normal
	abrasions
	lesions
	linea alba
	tattoos
	amalgam
	Stenson’s duct papilla
	xerostomia
chew/swallow	normal swallowing
	chew/swallow−not checked
	chew/swallow (cracker) w/o difficulty
	difficulty swallowing (water)
	chokes with swallowing
skin	texture
	normal
	xerosis
	hyperkeratosis pilari
	seborrhea
	eczematous rash
	color
	normal
	brown (acanthosis nigricans)
	purple (ecchymosis)
	hair
	normal
	swan neck hair (scurvy)
	lesions
	normal
	acne vulgaris
	keratosis
	cancers
	basal cell
	squamous cell
	melanoma
	poor wound healing
nails	shape
	color
	texture
	growth pattern changes
neurological exam	monofilament (5.07/10 gm)
	vibratory sense (128 hz)
	balance
	standing (eyes closed)
	single leg stand (eyes closed)
	motor: timed up and go
	smell test (cranial nerve 1)
	taste test (bitter) (cranial nerves, 7, 9, 10)

**Table 2:** Baseline characteristics of participants in LIFEHOUSE. Body mass index (BMI).

Descriptive	Total (*n* = 369)	Corporate/Sales (*n* = 257)	Manufacturing (*n* = 112)
age (year)	42.3 ± 10.9	42.8 ± 10.1	41.4 ± 12.5
BMI (kg/m^2^)	27.4 ± 6.0	26.5 ± 5.1	30.5 ± 7.4 *
BMI (kg/m^2^) female	26.5 ± 6.0	25.7 ± 5.4	29.5 ± 7.0 *
BMI (kg/m^2^) male	29.4 ± 5.7	28.4 ± 4.1	31.7 ± 7.8 *
waist circumference (cm)	91.4 ± 15.5	88.6 ± 14.0	99.3 ± 16.8 *
waist circumference (cm) female	86.6 ± 14.2	84.1 ± 13.0	94.0 ± 15.5 *
waist circumference (cm) male	100.1 ± 14.0	94.5 ± 12	108.2 ± 15.0 *
sex (% female)	242 (65.6%)	175 (68.1%)	67 (59.8%)
ethnicity/race			
African American	14 (6.2%)	9 (5%)	5 (10.9%)
Asian	25 (11%)	21 (11.6%)	4 (8.7%)
White	147 (64.8%)	120 (66.3%)	27 (58.7%)
Hispanic	27 (11.9%)	17 (9.4%)	10 (21.7%)
Native American	8 (3.5%)	8 (4.4%)	
other	6 (2.6%)	6 (3.3)	

* *p* < 0.001.

## Data Availability

Data is contained within the article.
